# Supplementary material for: Predicting performance of naïve migratory animals, from many wrongs to self-correction
Source: Commun Biol. 2022 Oct 4;5:1058. doi: 10.1038/s42003-022-03995-5 (PMC9532420; doi:10.1038/s42003-022-03995-5)
Supplement: Supplementary file 2 — Supplementary Information [file 42003_2022_3995_MOESM2_ESM.pdf]

Supplementary Information

Predicting performance of naïve migratory animals, from many wrongs to self-correction:

James D. McLaren[1]*, Heiko Schmaljohann[2,3], Bernd Blasius[1,4]

[1] Institute for Chemistry and Biology of the Marine Environment (ICBM), University of Oldenburg; 26129 Oldenburg, Germany.

[2] Institute for Biology and Environmental Sciences (IBU), Carl von Ossietzky University of Oldenburg, 26129 Oldenburg, Germany.

[3] Institute of Avian Research, 26386 Wilhelmshaven, Germany.

[4] Helmholtz Institute for Functional Marine Biodiversity (HIFMB), University of Oldenburg; 26129 Oldenburg, Germany.

*Corresponding author. Email: james.mclaren@uol.de

Supplementary Note 1

Sequences of von Mises headings

Compass and flight-step errors were simulated using a von Mises distribution, defined by an angular "concentration" parameter, κ, analogous to the reciprocal of variance in headings:

$$p(\alpha|\bar{\alpha},\kappa) = \frac{1}{2\pi I_0(\kappa)} e^{\kappa\cos(\alpha-\bar{\alpha})},$$

where I_0 is the modified Bessel function of the first kind and order zero[1]. These can be readily computed (we used MATLAB package vmrand, Dylan Muir). However, to estimate expected migratory performance, it is desirable to quantify effective precision across many flight-steps accounting for several sources of error. Unfortunately, unlike sums of normal variables, circular random errors do not sum in a scale-free way, or necessarily even follow the same distribution as their components[1,2]. However, for sufficiently small concentrations, κ , von Mises samples are similar to normally sampled variables[1] with circular standard deviations of

$$\sigma = 1/\sqrt{\kappa}\,. \qquad \text{(S1)}$$

Supplementary Fig. 1a (with triangle symbols colour-coded to σ) illustrates that Eq. S1 is reasonable for σ < 30°. Within this limitation, we can also quantify effective flight-step errors based on several within-step sources of error, e.g., cue transfers, we can apply the normal relations for the sum of two variables ($\sigma_{A+B} = \sqrt{\sigma_A^2 + \sigma_B^2}$), and for the average of *m* variables of uniform standard deviation ($\sigma_m = \sigma/\sqrt{m}$) to cue detection followed by *M* cue maintenance events[1]:

$$\sigma_{step} \approx \begin{cases} \sigma/\sqrt{M+1}\,, & \text{no cue transfer} \\ \sigma\sqrt{2+M^{-1}}, & \text{with cue transfer} \end{cases} \qquad \text{(S2)}$$

Supplementary Fig. 1a (circular symbols) illustrates, for *M* = 7 maintenance events (e.g., hourly re-determination during an 8-hour flight) that the flight-step precision is also a reasonable

approximation of actual standard circular deviation for compass precision σ < 30°. Eq. S2 further indicates that cue-maintenance reduces expected stepwise errors for non-transferred flight (main Fig. 1b, Supplementary Fig. 1b), but not so for flight with cue transfers (main Fig. 1c).

We can analogously estimate effective standard error after N steps for a single individual,

$$\sigma_N \cong \sigma_{step}/\sqrt{N}, \qquad \text{(S3)}$$

or within a migratory population, considering both within-individual effective error following the expected number of steps, $\widehat{N}$, and between-individual variability in preferred (inherited) headings, σ_{ind}:

$$\sigma_{pop} \cong \sqrt{\sigma_{ind}{}^2 + \sigma_{step}{}^2/\widehat{N}}.$$

The above equation reflects the importance of relatively low between-individual variability[3].

Supplementary Note 2

Migratory performance on a plane

Performance (arrival probability) of independent stepwise planar movement to a (circular) goal area of radius R_{goal} will approximate a cumulative normal distribution (erf function)[1], based on the breadth of successful angles, angular concentration in headings, and expected number of steps[4,5]. For long-distance migration, successful angles follow the goal-area breadth (Fig. 1, Table 1), since $\beta = R_{goal}/R_{mig} \cong \tan^{-1}(R_{goal}/R_{mig})$. Assuming uniform population headings and applying equation (S3) and the Central Limit Theorem for large numbers of steps[1], a first planar approximation to sufficiently directionally accurate migration is

$$\hat{p}_{\beta,\widehat{N}} \approx p\left(\left|\left(\frac{1}{\widehat{N}}\sum_{i=1}^{\widehat{N}} \alpha_i\right) - \bar{\alpha}\right| \leq \beta\right) \approx erf\left(\frac{\beta}{\sqrt{2}\,\sigma_{step}/\sqrt{\widehat{N}}}\right), \qquad \text{(S4)}$$

where $\widehat{N} = N_0 \cdot I_1(\kappa_{step})/I_0(\kappa_{step})$ is the expected number of steps, $\kappa_{step} \cong \sigma_{step}{}^{-2}$ (Eq. S1), and

$$N_0 = (R_{mig} - R_{goal})/R_{step} \qquad \text{(S5)}$$

is the minimum (error-free) number of steps to reach the closest edge of the goal area. From Eqs. S4-5 we see that within the planar and normal limit, i.e., high stepwise concentrations, κ_{step}, performance roughly follows the "length-adjusted goal breadth", i.e., Eq. 3 (main text):

$$\beta_{adj} = \beta\sqrt{N_0} = \beta\sqrt{(R_{mig} - R_{goal})/R_{step}}\,.$$

Seasonal migration constraints

In assessing performance, we also accounted for seasonal migration constraints via a population-specific maximum number of steps, N_{max} (Table 2; this became significant for the longest-distance simulations with large magnitudes equivalent errors). Using the Central Limit Theorem and known properties of sums of cosines $C_j(N) = \frac{1}{N}\sum_{i=1}^{N}\cos(j \cdot \alpha_i)$ [30,59], this is

$$p_{\emptyset,N_{max}} \cong p\left(R_{step}\sum_{i=1}^{N_{max}}\cos(\alpha_i) \geq R_{mig}\cos\bar{\alpha}\right) \cong \frac{1}{2}\left[1 - erf\left(\left(\frac{N_0}{N_{max}} - \frac{I_1(\kappa)}{I_0(\kappa)}\right)\cdot\frac{\cos\bar{\alpha}}{\sigma_C\sqrt{2}}\right)\right], \text{(S6)}$$

where $E\left[\left(C_j|\bar{\alpha}\right)\right] = \cos(j\bar{\alpha}) \cdot I_j\left(\kappa_{step}\right)/I_0\left(\kappa_{step}\right)$, and

$\sigma_C^2 = Var\left((C_1|\bar{\alpha})\right) = \frac{1}{2}\cdot(1 + E[C_2] - 2E[C_1]^2)$. Eq. S6 looks daunting, but can be readily computed (to calculate Bessel function values, we used MATLAB function besseli).

Supplementary Note 3

Efficient formula for sunset azimuth

Here we derive an equation for sunset azimuth, which aids interpretation and can speed up simulations involving large numbers of modelled individuals. The sun's zenith (angle relative to vertical, γ_s), is the inverse of solar elevation, i.e., $\gamma_s = 90^\circ$ at sunset. Solar zenith can be calculated using

$$\cos\gamma_s = \sin\phi\sin\delta_s + \cos\emptyset\cos\delta_s\cos H,$$

where $\emptyset$ is latitude, δ_s is the solar declination, i.e., the angle between the tilt of the Earth's axis and the plane of its orbit around the sun (see below), and $H = 15° \cdot (h - 12)$ (degrees) is the solar hour, h = 12 representing local noon[6]. Sunsets occur at 90° solar zenith, i.e.,

$$\cos H = -\tan\phi \tan\delta_s. \qquad \text{(S7)}$$

Solar azimuth (measured clockwise from equatorward, i.e., clockwise from South in the Northern Hemisphere) can be calculated as

$$\cos(\theta_s) = -\frac{\sin\delta_s \cos\emptyset - \cos H \cos\delta_s \sin\phi}{\sin\gamma_s},$$

which, if there is a sunset, reduces, after substituting for H using Eq. S7, to Eq. 9 in the main text:

$$\theta_s = \cos^{-1}\left(\frac{-\sin\delta_s}{\cos\phi}\right),$$

with the minus signs arising from referencing a geographic South axis[6], with θ_s always positive since the sun sets Westward in both Hemispheres. If no sunset occurs, substituting H = 180° results in $\cos\theta_s = -1$, i.e., $\theta_s = 0°$ or 180°, indicating the sun being at its lowest due-North (in the Northern Hemisphere) during polar summer and its highest (closest to the horizon) due-South during polar winter. Hence, a general formula for sunset azimuth relative to geographic South (or, in the Southern Hemisphere, to geographic North) is

$$\theta_s = \begin{cases} \cos^{-1}(-\sin\delta_s/\cos\phi) \\ 180° \text{ (24-hour light)} \\ 0° \text{ (24-hour dark)} \end{cases}$$

When simulating compass courses, solar declination, δ_s, was calculated using a formulation, precise to within 0.01°, as used in the SunAlign package (https://web.archive.org/web/20120423120622/http://www.green-life-innovators.org/tiki-

index.php?page=ETIMSDEC.BAS+Equation+of+TIMe+and+Solar+DEClination), accounting for the number of days since winter solstice and the eccentricity of the Earth's orbit:

$$\sin\delta_s = \sin(-23.44°)\ \cos\left(\frac{360°}{365.24}(t_i + 10) - \frac{360°}{\pi}0.0167\sin\left(\frac{360°}{365.24}(t_i - 2)\right)\right),$$

where t_i is the day of year (beginning January 1st).

References

1. Mardia, K. V. Statistics of Directional Data. *Journal of the Royal Statistical Society. Series B (Methodological)* **37**, 349–393 (1975).
2. Hillen, T., J. Painter, K., C. Swan, A. & D. Murtha, A. Moments of von mises and fisher distributions and applications. *Mathematical Biosciences and Engineering* **14**, 673–694 (2017).
3. Thorup, K., Rabøl, J. & Erni, B. Estimating variation among individuals in migration direction. *Journal of Avian Biology* **38**, 182–189 (2007).
4. Mouritsen, H. & Mouritsen, O. A Mathematical Expectation Model for Bird Navigation based on the Clock-and-Compass Strategy. *Journal of Theoretical Biology* **207**, 283–291 (2000).
5. Alerstam, Thomas. Bird Migration Performance on the Basis of Flight Mechanics and Trigonometry. in *Biomechanics in animal behaviour* (eds. P. Domenici & R.W. Blake) (Oxford University Press, 2000).
6. Jenkins, A. The Sun's position in the sky. *Eur. J. Phys.* **34**, 633–652 (2013).
7. Alerstam, T. & Pettersson, S.-G. Orientation along great circles by migrating birds using a sun compass. *Journal of Theoretical Biology* **152**, 191–202 (1991).

Supplementary Fig. 1. Comparison of sums of von Mises headings with normal relationships.

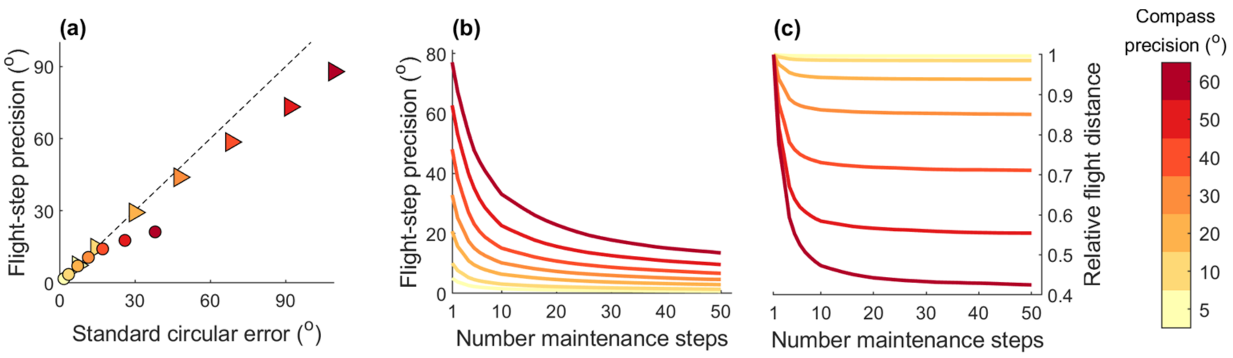

a Predicted precision (y-axis) of a flight-step including 7 in-flight maintenance events (e.g., hourly re-determination during an 8-hour flight) based on summation rules for normal headings (Eq. S2 and see Supplementary Note 1), versus (x-axis) actual standard circular deviations. Filled circles represent single-cue flight-steps and filled triangles represent cue-transferred steps, with colours depicting compass precision, $\sigma = 1/\sqrt{\kappa}$ (governing each detection, transfer and maintenance event), calculated using Eq. S1. **b** Resultant flight-step precision, and **c** relative stepwise flight distance (here, in the absence of cue transfers) as a function of the number of maintenance events, colour-coded to compass precision. Based on numerical simulations of 10,000 von Mises headings using MATLAB package vmrand (Dylan Muir).

Supplementary Fig. 2. Compass course trajectories of remaining species.

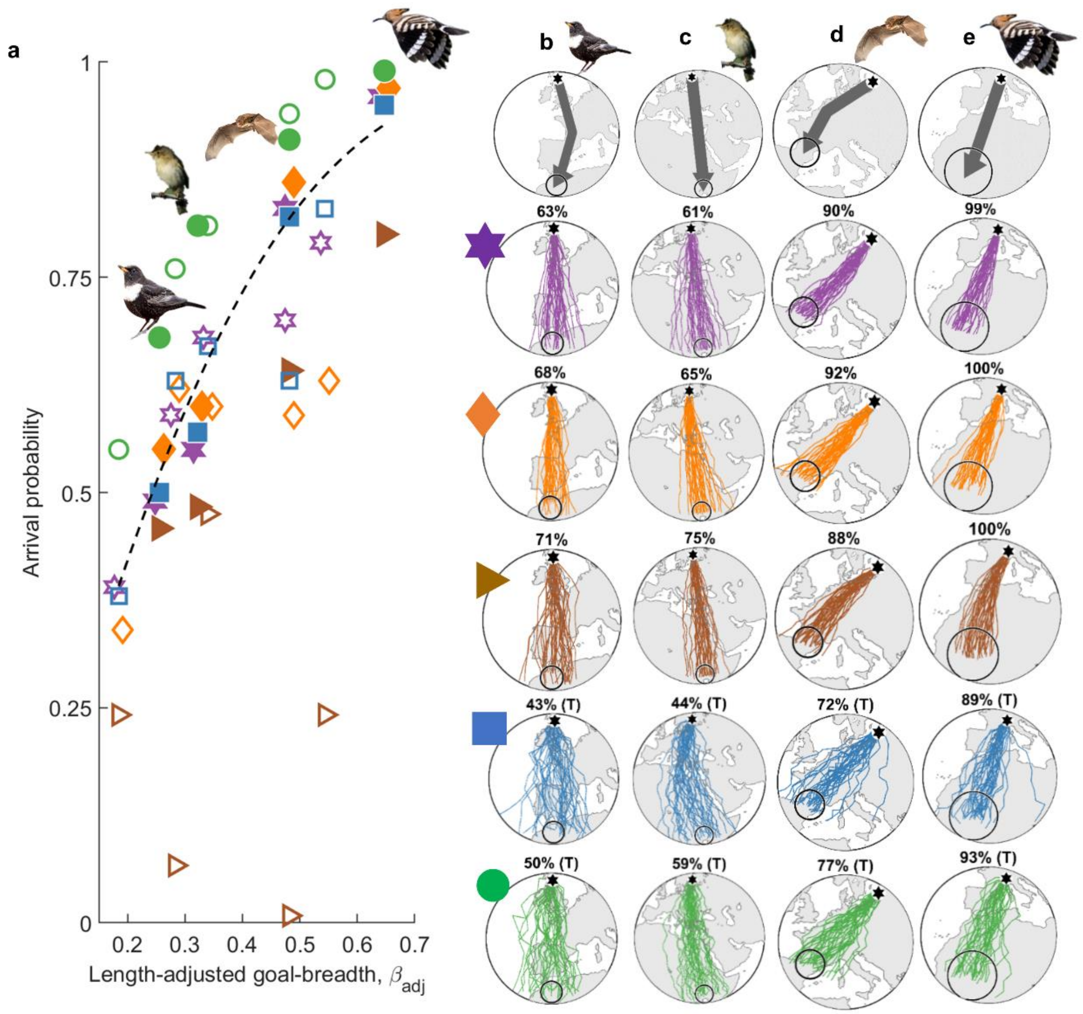

a Analogous to Fig. 4a, performance of simulated compass courses, assuming 20° directional precision among flight steps, of (solid symbols, left to right) night-migratory Ring Ouzel (Turdus torquatus), marsh warbler (*Acrocephalus palustris*), Nathusius bat (*Pipistrellus nathusii*) and Euraian hoopoe (*Upupa epops*) vs. length-adjusted goal-breadth, β_{adj} (main text Eq. 3), with open symbols representing species depicted in Fig. 4, and dashed line depicting expected performance in the normal planar limit (Eq. 16). **b-e** Randomly-selected trajectories assuming biologically-relevant error (see Fig. 4b-f), modelled after known routes (grey arrows) of **b** ring ouzel, **c** marsh warbler, **d** Nathusius bat and **e** Eurasian hoopoe, following geographic loxodromes (i.e., star compass courses, purple hexagons and tracks), geomagnetic loxodromes (orange diamonds), magnetoclinic courses (brown squares), fixed sun compass courses (blue circles) and time-compensated sun compass (TCSC) courses (green hexagons). Known migration routes, from natal grounds (black hexagons) to natural goal areas (open circles), are depicted by grey arrows (great circles appear as straight lines in the stereographic projection). Performance (%) and, where applicable, also cue-transferred courses ("T") are depicted above each panel. Photos by **a** P. Gomez, **b** M. Szczepanek (both: https://creativecommons.org/licenses/by-sa/3.0), **c** C. Giese, and **d** Copyright © Albert Molenaar, via Observation.org.

Supplementary Fig. 3. Comparison among cue-transferred TCSC courses with different compass use during flights and during stopover.

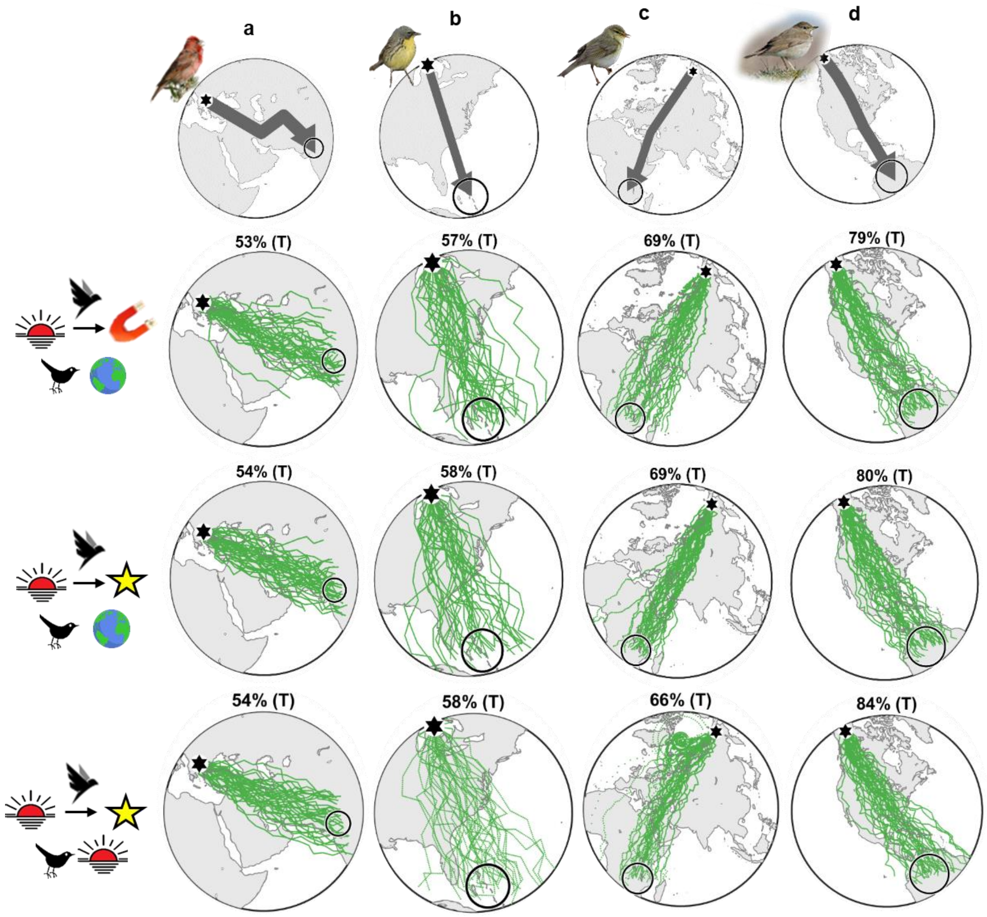

As in Fig. 4c-f, randomly-selected TCSC trajectories with biologically-relevant error (but here with 20º compass precision as well as 15º effective drift error) for known migration routes (**top row**) of **a** common rosefinch (*Carpodacus erythrinus*), **b** Kirtland's warbler (*Setophaga kirtlandii*), **c** willow warbler (*Phylloscopus trochilus*) and **d** gray-cheeked thrush (*Catharus minimus*) (see Table 2), based on cue transfer to (**second row**) a nocturnal geomagnetic compass and (**third-fourth rows**) transfer to a nocturnal star compass, where birds stopping over retain (**second-third rows**), as in Alerstam[7] and Fig. 4, geographic headings on arrival, and (**fourth row**) always retain TCSC headings from the first stopover night. Photos by **a** I. Shah (https://creativecommons.org/licenses/by-sa/4.0), **b** B. Majoros (https://creativecommons.org/licenses/by-sa/3.0/), **c** HS and **d** A. D'Entrement.

Supplementary Fig. 4. The spherical geometry factor.

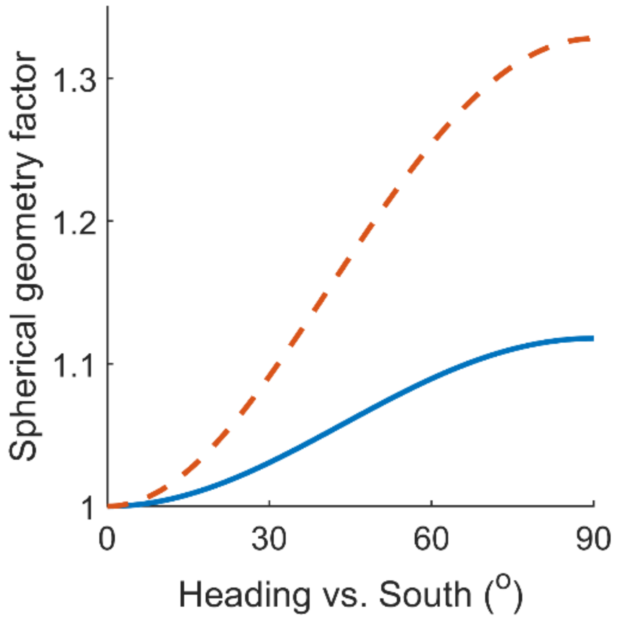

The spherical geometry factor (equation 18) vs. heading (clockwise from South) for long-distance migration (red dashed line, 65°N – 0º) and medium distance migration (solid blue line, 45ºN-25ºN).

Supplementary Fig. 5. Dependence of TCSC performance gain with compass precision and route and population specific factors.

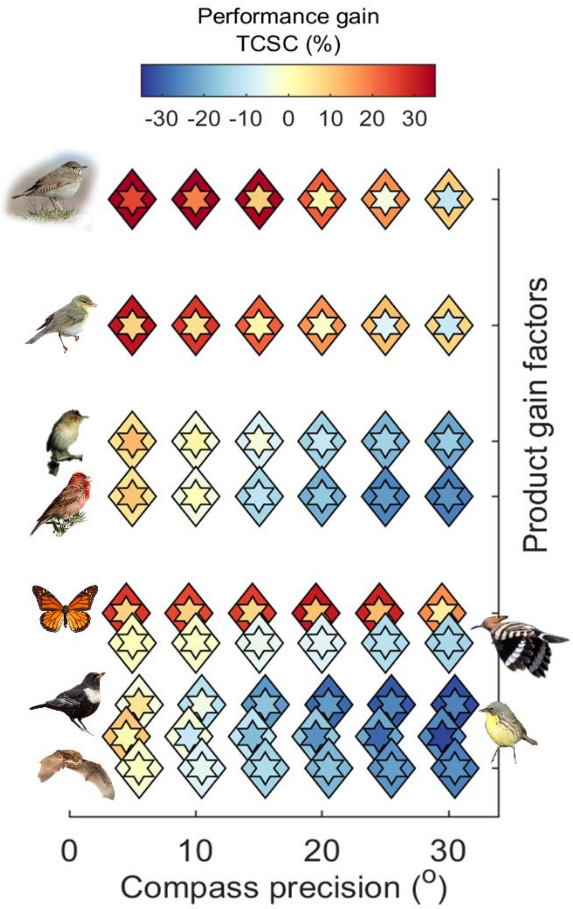

Relative performance gain (%) among species (see main text and Table 2) following TCSC courses over geographic loxodromes (e.g., star compass; colour-coded inner hexagons) and geomagnetic loxodromes (colour-coded outer diamonds) in biologically-relevant error scenarios versus (x-axis) compass precision in degrees, and (y-axis, shown in log-scale for visibility) the product of the three performance gain factors, i.e., the number of error-free flight-steps, spherical geometry factor (Suppl. Fig. 4) and flight-step distance (arc-degrees). Photos as per Supp. Figs. 2-3, and Monarch Butterfly by D. Descousens (https://creativecommons.org/licenses/by-sa/4.0/).

Supplementary Table 1.

AICc-selected parameter values of most parsimonious regression model (with lowest ΔAICc value) among model-selected regression based on route-optimized performance among species for each compass course.

**Compass course and adjusted R-squared**	**Parameter value and CI**	**p-value**
Geographic loxodrome $R^2_{adj} = 0.98$	g = 0.9 ± 0.3	0.002
	b_0= -0.02 ± 0.01	0.04
	s = 0.17 ± 0.03	10^{-5}
	ρ = (0)	not tested
Geomagnetic loxodrome $R^2_{adj} = 0.97$	g = 2.7 ± 0.3	10^{-11}
	b_0= 0 ± 0.01	0.9
	s = 0.15 ± 0.03	10^{-6}
	ρ = (0)	not tested
TCSC $R^2_{adj} = 0.98$	g = 0.8 ± 0.3	0.01
	b_0= 0.15 ± 0.01	10^{-14}
	s = 0.30 ± 0.03	10^{-11}
	ρ = 0.6 ± 0.2	0.001

Supplementary Table 2.

AICc-selected parameters (with ΔAICc < 2) among model-selected regression based on route-optimized performance among species for each compass course.

**Compass course and adjusted R-squared**	**Selected parameters**	**ΔAICc**
Geographic loxodrome	g, b_0, s	0
	b_0, s	0.23
Geomagnetic loxodrome	g, b_0, s	0
	g, s	0.01
TCSC	g, b_0, s, ρ	0
	b_0, s, ρ	0.80
